# Supplementary material for: Causal Relationship between Aging and Anorexia Nervosa: A White-Matter-Microstructure-Mediated Mendelian Randomization Analysis
Source: Biomedicines. 2024 Aug 16;12(8):1874. doi: 10.3390/biomedicines12081874 (PMC11351342; doi:10.3390/biomedicines12081874)
Supplement: Supplementary file 1 [file biomedicines-12-01874-s001.zip › Supplementary Figure/Figure S4/Singe SNP plot of MR analysis for TL to ubm-b-1977.pdf]

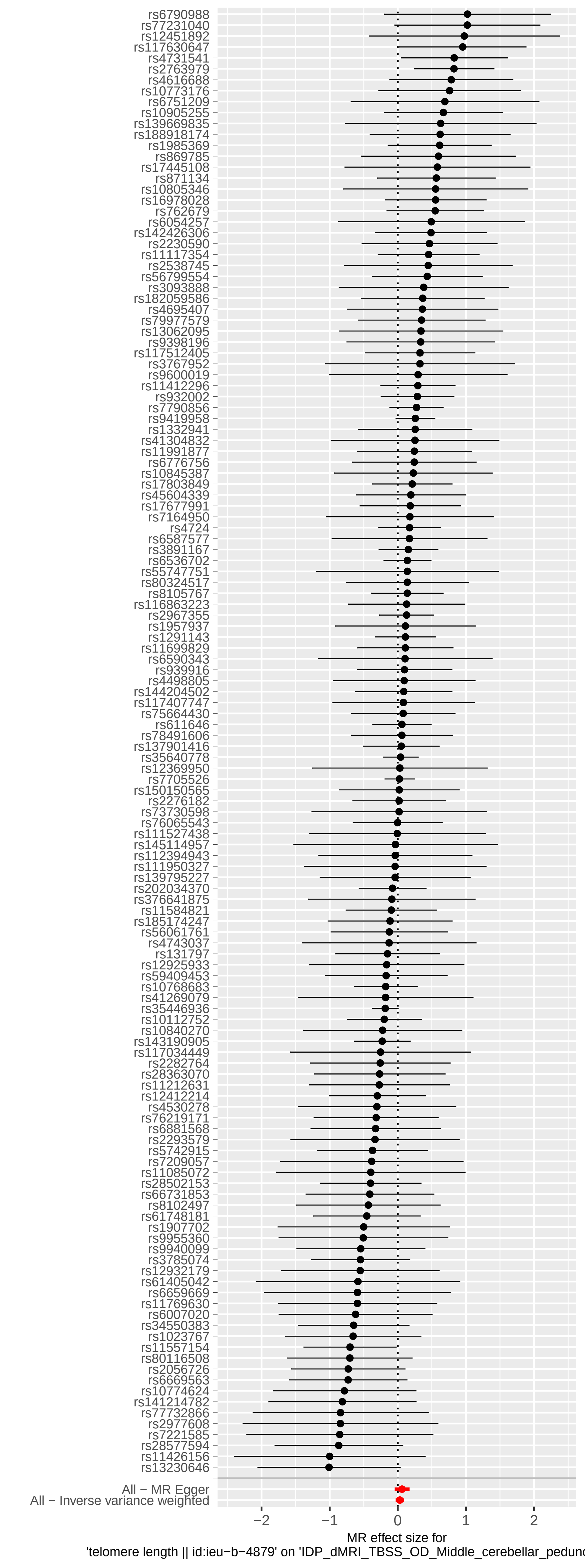
rs6790988
rs77231040
rs12451892
rs117630647
rs4731541
rs2763979
rs4616688
rs10773176
rs6751209
rs10905255
rs139669835
rs188918174
rs1985369
rs869785
rs17445108
rs871134
rs10805346
rs16978028
rs762679
rs6054257
rs142426306
rs2230590
rs11117354
rs2538745
rs56799554
rs3093888
rs182059586
rs4695407
rs79977579
rs13062095
rs9398196
rs117512405
rs3767952
rs9600019
rs11412296
rs932002
rs7790856
rs9419958
rs1332941
rs41304832
rs11991877
rs6776756
rs10845387
rs17803849
rs45604339
rs17677991
rs7164950
rs4724
rs6587577
rs3891167
rs6536702
rs55747751
rs80324517
rs8105767
rs116863223
rs2967355
rs1957937
rs1291143
rs11699829
rs6590343
rs939916
rs4498805
rs144204502
rs117407747
rs75664430
rs611646
rs78491606
rs137901416
rs35640778
rs12369950
rs7705526
rs150150565
rs2276182
rs73730598
rs76065543
rs111527438
rs145114957
rs112394943
rs111950327
rs139795227
rs202034370
rs376641875
rs11584821
rs185174247
rs56061761
rs4743037
rs131797
rs12925933
rs59409453
rs10768683
rs41269079
rs35446936
rs10112752
rs10840270
rs143190905
rs117034449
rs2282764
rs28363070
rs11212631
rs12412214
rs4530278
rs76219171
rs6881568
rs2293579
rs5742915
rs7209057
rs11085072
rs28502153
rs66731853
rs8102497
rs61748181
rs1907702
rs9955360
rs9940099
rs3785074
rs12932179
rs61405042
rs6659669
rs11769630
rs6007020
rs34550383
rs1023767
rs11557154
rs80116508
rs2056726
rs6669563
rs10774624
rs141214782
rs77732866
rs2977608
rs7221585
rs28577594
rs11426156
rs13230646
All − MR Egger
All − Inverse variance weighted
−2
−1
0
1
2
MR effect size for
'telomere length || id:ieu−b−4879' on 'IDP_dMRI_TBSS_OD_Middle_cerebellar_pedunc
